# A combination of *TERT* promoter mutation and *MGMT* methylation status predicts clinically relevant subgroups of newly diagnosed glioblastomas

**DOI:** 10.1186/s40478-016-0351-2

**Published:** 2016-08-08

**Authors:** Hideyuki Arita, Kai Yamasaki, Yuko Matsushita, Taishi Nakamura, Asanao Shimokawa, Hirokazu Takami, Shota Tanaka, Akitake Mukasa, Mitsuaki Shirahata, Saki Shimizu, Kaori Suzuki, Kuniaki Saito, Keiichi Kobayashi, Fumi Higuchi, Takeo Uzuka, Ryohei Otani, Kaoru Tamura, Kazutaka Sumita, Makoto Ohno, Yasuji Miyakita, Naoki Kagawa, Naoya Hashimoto, Ryusuke Hatae, Koji Yoshimoto, Naoki Shinojima, Hideo Nakamura, Yonehiro Kanemura, Yoshiko Okita, Manabu Kinoshita, Kenichi Ishibashi, Tomoko Shofuda, Yoshinori Kodama, Kanji Mori, Yusuke Tomogane, Junya Fukai, Koji Fujita, Yuzo Terakawa, Naohiro Tsuyuguchi, Shusuke Moriuchi, Masahiro Nonaka, Hiroyoshi Suzuki, Makoto Shibuya, Taketoshi Maehara, Nobuhito Saito, Motoo Nagane, Nobutaka Kawahara, Keisuke Ueki, Toshiki Yoshimine, Etsuo Miyaoka, Ryo Nishikawa, Takashi Komori, Yoshitaka Narita, Koichi Ichimura

**Affiliations:** 1 Division of Brain Tumor Translational Research, National Cancer Center Research Institute, 5-1-1 Tsukiji, Chuo-ku, Tokyo, 104-0045 Japan; 2 Department of Neurosurgery, Osaka University Graduate School of Medicine, Osaka, Japan; 3 Department of Pediatric Hematology and Oncology, Osaka City General Hospital, Osaka, Japan; 4 Department of Neurosurgery and Neuro-Oncology, National Cancer Center Hospital, Tokyo, Japan; 5 Department of Neurosurgery, Graduate School of Medicine, Yokohama City University, Yokohama, Japan; 6 Department of Mathematics, Faculty of Science, Tokyo University of Science, Tokyo, Japan; 7 Department of Neurosurgery, The University of Tokyo, Tokyo, Japan; 8 Department of Neuro-Oncology/Neurosurgery, Saitama Medical University International Medical Center, Saitama, Japan; 9 Department of Neurosurgery, Kyorin University Faculty of Medicine, Tokyo, Japan; 10 Department of Neurosurgery, Dokkyo Medical University, Tochigi, Japan; 11 Department of Neurosurgery, Tokyo Medical and Dental University, Tokyo, Japan; 12 Department of Neurosurgery, Kyushu University Graduate School of Medical Science, Fukuoka, Japan; 13 Department of Neurosurgery, Graduate School of Life Sciences, Kumamoto University, Kumamoto, Japan; 14 Division of Regenerative Medicine, Institute for Clinical Research, Osaka National Hospital, National Hospital Organization, Osaka, Japan; 15 Department of Neurosurgery, National Hospital Organization Osaka National Hospital, Osaka, Japan; 16 Department of Neurosurgery, Osaka Medical Center for Cancer and Cardiovascular Diseases, Osaka, Japan; 17 Department of Neurosurgery, Osaka City General Hospital, Osaka, Japan; 18 Division of Stem Cell Research, Institute for Clinical Research, Osaka National Hospital, National Hospital Organization, Osaka, Japan; 19 Central Laboratory and Surgical Pathology, Osaka National Hospital, National Hospital Organization, Osaka, Japan; 20 Department of Neurosurgery, Kansai Rosai Hospital, Hyogo, Japan; 21 Department of Neurosurgery, Hyogo College of Medicine, Hyogo, Japan; 22 Department of Neurological Surgery, Wakayama Medical University, Wakayama, Japan; 23 Department of Neurosurgery, Osaka City University Graduate School of Medicine, Osaka, Japan; 24 Department of Neurosurgery, Rinku General Medical Center, Izumisano, Osaka Japan; 25 Department of Pathology and Laboratory Medicine, National Hospital Organization, Sendai Medical Center, Sendai, Japan; 26 Central Laboratory, Hachioji Medical Center, Tokyo Medical University, Tokyo, Japan; 27 Department of Laboratory Medicine and Pathology (Neuropathology), Tokyo Metropolitan Neurological Hospital, Tokyo, Japan

**Keywords:** *TERT*, Molecular classification, Glioblastoma, Temozolomide, 1p19q, Glioma, *IDH1/2*, Prognostic factor

## Abstract

The prognostic impact of *TERT* mutations has been controversial in *IDH*-wild tumors, particularly in glioblastomas (GBM). The controversy may be attributable to presence of potential confounding factors such as *MGM*T methylation status or patients’ treatment. This study aimed to evaluate the impact of *TERT* status on patient outcome in association with various factors in a large series of adult diffuse gliomas. We analyzed a total of 951 adult diffuse gliomas from two cohorts (Cohort 1, *n* = 758; Cohort 2, *n* = 193) for *IDH1/2*, 1p/19q, and *TERT* promoter status. The combined *IDH/TERT* classification divided Cohort 1 into four molecular groups with distinct outcomes. The overall survival (OS) was the shortest in *IDH* wild-type/*TERT* mutated groups, which mostly consisted of GBMs (*P* < 0.0001). To investigate the association between *TERT* mutations and *MGMT* methylation on survival of patients with GBM, samples from a combined cohort of 453 *IDH*-wild-type GBM cases treated with radiation and temozolomide were analyzed. A multivariate Cox regression model revealed that the interaction between *TERT* and *MGMT* was significant for OS (*P* = 0.0064). Compared with *TERT* mutant-*MGMT* unmethylated GBMs, the hazard ratio (HR) for OS incorporating the interaction was the lowest in the *TERT* mutant-*MGMT* methylated GBM (HR, 0.266), followed by the *TERT* wild-type-*MGMT* methylated (HR, 0.317) and the *TERT* wild-type-*MGMT* unmethylated GBMs (HR, 0.542). Thus, patients with *TERT* mutant-*MGMT* unmethylated GBM have the poorest prognosis. Our findings suggest that a combination of *IDH*, *TERT*, and *MGMT* refines the classification of grade II-IV diffuse gliomas.

**Electronic supplementary material:**

The online version of this article (doi:10.1186/s40478-016-0351-2) contains supplementary material, which is available to authorized users.

## Introduction

Extensive genomic analyses have recently revealed that the biology of brain tumors, therefore patients’ clinical outcomes, is often determined by combinations of specific genetic and/or epigenetic alterations. The latest edition of the World Health Organization (WHO) Classification of Tumours of the Central Nervous System (revised 4th edition) incorporated molecular classification as a part of the integrated diagnosis, adding this to conventional histopathology and WHO grading [19]. This is a highly significant step in the diagnostic pathology, considering that the conventional histopathological diagnosis may suffer from morphological ambiguity and inter-observer discordance, most typically exemplified by oligoastrocytoma [33]. For molecular classification to best help standardize diagnoses, it is critical to test the efficacy of molecular markers to define specific entities.

Diffuse astrocytoma (DA) and anaplastic astrocytoma (AA) are best characterized by the presence of *IDH1/IDH2* mutations (“*IDH* mutation”), most often (but not always) accompanied by mutations in *TP53* and *ATRX* [12, 17, 31]. It has been suggested that the *IDH* mutation is a founder mutation that precedes *TP53* and *ATRX* mutations [31]. The presence of *IDH* mutation is associated with significantly longer overall survival in astrocytoma patients diagnosed, according to the WHO 2007 Classification [11, 36]. Oligodendroglioma is defined by the concurrent deletions of entire 1p/19q (“1p/19q codeletion”), which is invariably accompanied by *IDH* mutation. The 1p/19q codeletion is caused by an unbalanced t(1;19)(q10;p10) translocation resulting in total loss of one copy of 1p and 19q [8]. Mutations of *FUBP1* (1p31.1) and/or *CIC* (19q13.2) are found in 52–66 % of oligodendrogliomas [4, 12]. The spatial/temporal distribution of *FUBP1/CIC* mutations may be heterogeneous, whereas the 1p/19q codeletion is homogeneously found within the tumor tissue [31], and a considerable number of 1p/19q codeleted tumors have no mutations to *FUBP1/CIC* [31]. Thus, astrocytomas and oligodendrogliomas will be diagnosed based on molecular characterization of the *IDH* and 1p/19q statuses; diffuse astrocytomas are defined by the presence of *IDH1/IDH2* mutations without 1p/19q codeletion, whereas the diagnosis of oligodendrogliomas requires the presence of both *IDH* mutation and 1p/19q codeletion. Molecular classification of *IDH*-wildtype gliomas is somewhat elusive. The great majority of primary GBMs are *IDH* wild-type. It has been suggested that most astrocytomas with wild-type *IDH* may resolve into other tumor entities, mostly glioblastomas (GBMs) [28]. For better definition of GBMs in *IDH*-wild-type tumors, further classification of molecular markers is needed.

*TERT* promoter mutations are very common in GBMs and oligodendroglial tumors [1, 14]. Mutations occur at either of the two hotspots (conventionally referred to as C228T and C250T for their chromosomal coordinates in the hg19 assembly) in a mutually exclusive manner. The mutations create *de novo* GA Binding Protein Transcription Factor Alpha Subunit (GABPA) binding sites [3], causing an increase in *TERT* mRNA transcription in GBM [1, 3], a mechanism that would lead to telomerase upregulation and telomere elongation. The *TERT* promoter mutations almost always coincide with *IDH* mutations and 1p/19q codeletion in oligodendrogliomas, whereas a combination of *TERT* mutation and wild-type *IDH* is the most common genotype observed in GBM. These findings suggest that the combination of *IDH* and *TERT* mutations may be useful to define glioma subclasses. The prognostic impact of *TERT* mutation in diffuse gliomas appears to be bivalent, unlike *IDH* mutation or 1p/19q codeletion. Concurrent mutations of *TERT* and *IDH* predict good prognosis, as an alternative hallmark of oligodendroglioma, whereas *TERT* promoter mutation with wild-type *IDH* tends to be associated with poor prognosis, although its use in predicting outcomes in GBM is controversial [1, 6, 13, 15, 26, 30]. One of the potential confounding factors in the prognostication of GBM is the methylation status of the *MGMT* promoter. *MGMT* promoter methylation (“*MGMT* methylation”) is a well-established prognostic marker for primary GBM and a predictive marker for the response to temozolomide in elderly GBM [9, 22, 34].

In this study, we examined the utility of molecular classification based on the *IDH* and *TERT* statuses to predict clinical courses of patients in association with various clinical factors, histological diagnosis, and grading in a large series of newly diagnosed WHO grade II-IV adult gliomas. We specifically focused on the potential interaction between *MGMT* promoter methylation and *TERT* mutational statuses to further refine the clinical value of molecular diagnosis. We found that *TERT* mutation identifies a subset of GBM patients who are most resistant to the conventional radiochemotherapy when *MGMT* is unmethylated.

## Materials and methods

### Patient selection

**Fig. 1:**
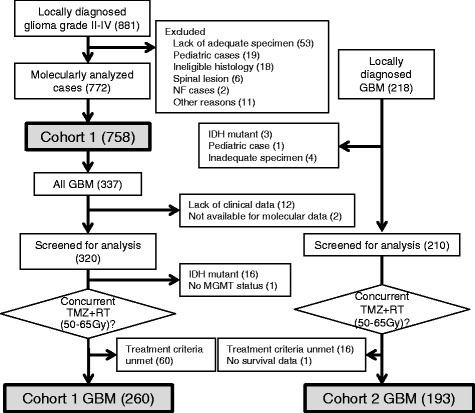
Flowchart of patient selection. For Cohort 1, 758 out of 881 Grade II-IV cases collected met the eligibility criteria and were analyzed for the prognostic impact of *IDH* and *TERT* status in adult diffuse gliomas. From Cohort 1, 260 GBM patients concurrently treated with TMZ and RT were further selected (Cohort 1 GBM). For Cohort 2, 193 *IDH* wild-type GBM cases treated with TMZ plus RT were selected (Cohort 2 GBM). Cohort 1 GBM and Cohort 2 GBM were analyzed for the influences of *TERT* and *MGMT* status on survival. *GBM*, glioblastoma; *NF*, neurofibromatosis; *RT*, radiation therapy; *TMZ*, temozolomide

Two cohorts were collected in this study. Cohort 1 was formed to evaluate the prognostic impact of molecular classification based on *IDH* and *TERT* statuses in adult diffuse gliomas. The inclusion criteria for the Cohort 1 were as follows: 18 years of age or older, histological diagnosis of grade II-IV diffuse glioma originating in the cranium, genomic DNA available for molecular analysis (extracted from frozen tumor tissues taken at the time of the initial surgery), and clinical data available for survival analysis. Out of 881 cases initially collected from 13 institutions in Japan, 758 cases met the criteria and enrolled in the study as Cohort 1 (the diagram of case selection is shown in Fig. 1). To further analyze the impact of *TERT* promoter mutation and *MGMT* methylation status on survival, Cohort 2 was collected as an independent set of locally diagnosed GBM. The inclusion criteria for the Cohort 2 were: 18 years of age or older, histological diagnosis of GBM, treatment with local irradiation of 50 – 65 Gy and concurrent chemotherapy with temozolomide after initial surgery, absence of clinically apparent preceding lower grade gliomas, no *IDH1*/2 mutations, clinical data available for survival analysis, and genomic DNA available for molecular analysis (extracted from frozen tissues taken at the time of the initial surgery). Out of 218 cases collected from four institutions in Japan, 193 cases met the criteria described above and were enrolled in Cohort 2. Clinical data collected from each institution included the detailed information as follows: age, sex, preoperative Karnofsky Performance status (KPS), extent of resection, radiation dose, and chemotherapeutic regimen in initial treatment. The study was approved by the Institutional Review Board (IRB) at National Cancer Center (No. 2013–042) and the corresponding local IRB of the participating centers.

### Central pathology review

All cases of Cohort 1 were subjected to central pathology review by three senior neuropathologists (T.K., M.S., and H.S.). Histological diagnosis was made as a consensus of the 3 pathologists based on the fourth edition of WHO Classification (WHO 2007) [18]. Although the cases of the Cohort 2 were not subjected to these review procedures, the local diagnoses were based on the WHO 2007 criteria, similar to Cohort 1.

### Molecular analysis

Tumor DNA was extracted from frozen tumor tissues for all cases using a DNeasy Blood & Tissue Kit (Qiagen, Tokyo, Japan). The details of genetic analysis, including PCR and sequencing for each gene status, are described in the Additional file 1: Supplementary materials. The presence of hotspot mutations in *IDH1* (R132) and *IDH2* (R172) was assessed by pyrosequencing for all cases included in this study, as previously reported [2]. The two mutation hotspots in the *TERT* promoter were analyzed in all tumors by Sanger sequencing and/or pyrosequencing, as previously reported [1]. The mutation hotspots at codons 27 and 34 of *H3F3A* and at codon 600 of *BRAF* were analyzed by Sanger sequencing and/or pyrosequencing. The copy number statuses of 1p and 19q were determined by multiplex ligation-dependent probe amplification (MLPA), Microarray-based comparative genomic hybridization (aCGH) or microsatellite analysis [1, 24, 32]. The copy number status of *CDKN2A* was also assessed using MLPA or aCGH [1]. The methylation status of the *MGMT* promoter was analyzed by pyrosequencing after bisulfite modification of genomic DNA extracted from tumor specimens as described [25], with some modifications in the thermal cycling conditions. Based on an outcome-based study to determine an optimal cutoff to judge *MGMT* promoter methylation in a series of 276 newly diagnosed GBMs, we used a cut-off of ≥ 16 % for *MGMT* methylation. The details of this study will be described elsewhere (Ichimura, manuscript in preparation).

### Statistical analysis

Statistical analysis was performed using an SAS package and JMP version 10 (SAS Institute, Cary, NC, USA). Categorized data were compared between subgroups using the chi-square test. The comparison for age distribution was examined with the Student’s t test. The survival data were analyzed with the log-rank test and univariate and multivariate Cox regression analyses. Stepwise procedure was used in multivariate Cox regression. In multivariate Cox proportional hazard models for GBM cases including *TERT* and *MGMT*, a term of interaction between them was also incorporated. A P-value < 0.05 was considered significant in statistical analyses.

## Results

### Molecular classification based on *IDH* and *TERT* defines distinct subgroups of adult gliomas in Cohort 1

All 758 tumor samples from Cohort 1 were screened for mutations in *IDH1/2* and *TERT* hotspots and the copy number status of 1p/19q. The data were determined for all cases but one, in which the 1p/19q status was not determined. The frequencies of each molecular status for each histology are shown in Table 1. The molecular status, patient background, and clinical information for each case are provided in Additional file 2: Table S1. *IDH1/2* mutations were present in 38 % of tumors, mostly a c.395G > A transition in *IDH1* (R132H; 274/286, 96 %). *TERT* promoter mutations were observed in 51 % (390/758) of tumors. *IDH1/2* mutations were common in grade II-III gliomas (38–96 %), whereas *TERT* promoter mutations were frequently observed in oligodendrogliomas (74–83 %) and GBMs (58 %).

**Table 1:** Mutation types of *IDH* and *TERT* in each histological type in Cohort 1 and 2, each

		Cohort 1								Cohort 2
Histology		DA	AA	GBM	OA	AOA	OL	AO	All	GBM^a^
Total		63	106	337	67	103	47	35	758	193
*IDH1/2* n(%)	R132H	36 (57)	38 (36)	16 (5)	56 (84)	59 (57)	42 (89)	27 (77)	274 (36)	0 (0)
	Other mutation	2 (3)	2 (2)	0 (0)	2 (3)	2 (2)	3 (6)	1 (3)	12 (2)	0 (0)
	All mutation	38 (60)	40 (38)	16 (5)	58 (87)	61 (59)	45 (96)	28 (80)	286 (38)	0 (0)
*TERT* n(%)	C228T	12 (19)	29 27)	136 (40)	27 (40)	30 (29)	27 (57)	17 (49)	278 (37)	94 (49)
	C250T	5 (8)	6 (6)	58 (17)	6 (9)	15 (15)	12 (26)	9 (26)	111 15)	21 (11)
	C228T&C250T	1 (2)	0 (0)	0 (0)	0 (0)	0 (0)	0 (0)	0 (0)	1 (0.1)	0 (0)
	All mutation	18 (29)	35 (33)	194 (58)	33 (49)	45 (44)	39 (83)	26 (74)	390 (51)	115 (60)
1p/19q n(%)	Total 1p/19q loss	9/63 (14)	6/106 (6)	6/336 (2)	27/67 (40)	32/103 (31)	41/47 (87)	27/35 (77)	148/757 (20)	N/A
*MGMT* n(%)	Methylated	35/62 (56)	45/106 (42)	126/336 (38)	44/66 (67)	67/103 (65)	39/47 (83)	30/35 (86)	386/755 (51)	66/193 (34)
*H3.3*/*H3.1* n(%)	K27M	2/34 (6)	6/71 (9)	12/316 (4)	0/20 (0)	1/47 (2)	0/12 (0)	0/10 (0)	21/510 (4)	0/193 (0)
	Other mutation	0/34 (0)	0/71 (0)	0/316 (0)	0/20 (0)	1/47 (2)	1/12 (8)	0/10 (0)	2/510 (0.4)	0/193 (0)
*BRAF* (V600) n(%)	V600E	1/34 (3)	3/70 (4)	8/313 (3)	0/20 (0)	0/47 (0)	0/12 (0)	0/10 (0)	12/506 (2)	4/193 (2)
*CDKN2A* n(%)	Homozygous Del	3/48 (6)	13/74 (18)	86/229 (37)	0/42 (0)	11/64 (17)	0/28 (0)	3/20 (15)	116/505 (23)	N/A
	Heterozygous Del	2/48 (4)	15/74 (20)	40/229 (17)	1/42 (2)	9/64 (14)	2/28 (7)	3/20 (15)	72/505 (14)	N/A

^a^ *IDH1/2* mutated cases were excluded for Cohort 2 (see Materials and methods in the manuscript)

*AA* anaplastic astrocytoma, *AO* anaplastic oligodendroglioma, *AOA* anaplastic oligoastrocytoma, *DA* diffuse astrocytoma, *Del* Deletion, *GBM* glioblastoma, *N/A* not available, *OA* oligoastrocytoma, *OL* oligodendroglioma

**Fig. 2:**
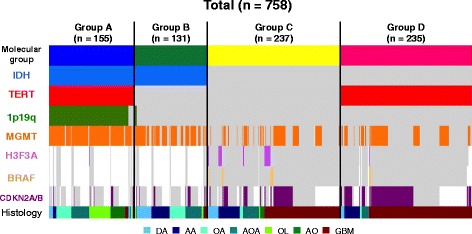
A diagram of molecular classification of Cohort 1. All 758 tumors in Cohort 1 are sorted according to their molecular classification based on their *IDH* and *TERT* statuses. The mutation statuses of *IDH*, *TERT*, *H3F3A*, the copy number statuses of 1p/19q and *CDKN2A/B BRAF*, and *MGMT* methylation are shown. Centrally reviewed histology is indicated at the bottom. Gray or colored cells indicate absence or presence of alterations, respectively. Blank cells denote no data. Group A, *IDH* mutated-*TERT* mutated; Group B, *IDH* mutated-*TERT* wild-type; Group C, *IDH* wild-type-*TERT* wild-type; Group D, *IDH* mutated-*TERT* mutated. *AA*, anaplastic astrocytoma; *AO*, anaplastic oligodendroglioma; *AOA*, anaplastic oligoastrocytoma; *DA*, diffuse astrocytoma; *Del*, Deletion; *GBM*, glioblastoma; *OA*, oligoastrocytoma; *OL*, oligodendroglioma

The combined *IDH/TERT* classification divided the Cohort 1 into four molecular groups, each showing distinct patient characteristics, histology, or clinical outcome. The patient backgrounds and molecular status of each group are summarized in Table 2 and Fig. 2. The group with mutations in both *IDH* and *TERT* (Group A) mainly consisted of oligodendrogliomas or oligoastrocytomas (82 %). The group with mutation in *IDH* but not *TERT* (Group B) mostly consisted of astrocytomas and grade II-III oligoastrocytomas. The group with no detectable *IDH* or *TERT* hotspot mutations (Group C) included all types of histology, GBM being the most common (57 %). Tumors exhibiting *TERT* mutation with wild-type *IDH* (Group D) were mostly GBMs (79 %) or AAs (12 %).

**Table 2:** Patient background and molecular status of Cohort 1

	All	Group A^a^	Group B^a^	Group C^a^	Group D^a^	p-value^b^
Total	758	155	131	237	235	
Mean Age at diagnosis(year)	53.7	44.7	41.7	56.7	63.4	<0.0001
Sex M/F	426/332	90/65	75/56	134/103	127/108	0.87
Surgery n (%)						
Biopsy only	121 (16)	16 (10)	19 (15)	49 (21)	37 (16)	0.05
KPS n (%)						
100	160 (21)	66 (43)	51 (40)	26 (11)	17 (7)	<0.0001
90	268 (35)	63 (41)	58 (45)	83 (35)	64 (27)	
80	126 (17)	13 (8)	7 (5)	43 (18)	63 (27)	
70	94 (12)	6 (4)	8 (6)	38 (16)	42 (18)	
− 60	107 (14)	7 (5)	4 (3)	47 (20)	49 (21)	
Adjuvant therapy n (%)						
CRT	559/748 (75)	83/150 (55)	70/131 (53)	201/235 (86)	205/232 (88)	<0.0001
Chemo only	58/748 (8)	32/150 (21)	5/131 (4)	7/235 (3)	14/232 (6)	
RT only	43/748 (6)	4/150 (3)	16/131 (12)	13/235 (6)	10/232 (4)	
None	88/748 (12)	31/150 (21)	40/131 (31)	14/235 (6)	3/232 (1)	
Location n(%)						
Cerebrum (with frontal involvement)	370/756 (49)	119/154 (77)	82/131 (63)	86/237 (36)	83/234 (35)	<0.0001
Cerebrum (other)	348/756 (47)	34/154 (23)	48/131 (37)	122/237(53)	144/234 (63)	
Thalamus	25/756 (3)	1/154 (0.7)	0/131 (0)	18/237 (8)	6/234 (3)	
Infratentorium	13/756 (2)	0/154 (0)	1/131 (0.8)	11/237 (5)	1/234 (0.4)	
Histology n (%)						
DA	63 (8)	12 (8)	26 (20)	19 (8)	6 (3)	<0.0001
AA	106 (14)	7 (5)	33 (25)	38 (16)	28 (12)	
GBM	337 (44)	9 (6)	7 (5)	136 (57)	185 (79)	
OA	67 (9)	30 (19)	28 (21)	6 (3)	3 (1)	
AOA	103 (14)	32 (21)	29 (22)	29 (12)	13 (6)	
OL	47 (6)	39 (25)	6 (5)	2 (1)	0 (0)	
AO	35 (5)	26 (17)	2 (2)	7 (3)	0 (0)	
Molecular background n(%)						
Total 1p/19q loss	148/757 (20)	144/155 (93)	4/131 (3)	0/237 (0)	0/234 (0)	<0.0001
*MGMT* methylation	386/755 (51)	143/155 (92)	88/129 (68)	72/236 (31)	83/235 (35)	<0.0001
*H3.3/H3.1* mutation	23/510 (5)	1/31 (3)	0/21 (0)	21/233 (9)	1/235 (0.4)	0.0001
*BRAF* V600E	12/506 (2)	0/31 (0)	0/21 (0)	9/233 (4)	3/221 (1)	0.21
*CDKN2A*						
Homozygous Del	116/505 (23)	4/89 (4)	7/86 (8)	37/163 (23)	68/167 (41)	<0.0001
Heterozygous Del	72/505 (14)	5/89 (6)	6/86 (7)	22/163 (13)	39/167 (23)	

*AA* anaplastic astrocytoma, *AO* anaplastic oligodendroglioma, *AOA* anaplastic oligoastrocytoma, *Chemo* Chemotherapy, *CRT* chemoradiotherapy, *DA* diffuse astrocytoma, *Del* Deletion, *F* Female, *GBM* glioblastoma, *KPS* Karnofsky Performance Status, *M* Male, *N/A* not available, *OA* oligoastrocytoma, *OL* oligodendroglioma, *RT* radiation therapy

^a^ *Group A* IDH mutated-TERT mutated, *Group B* IDH mutated-TERT wid-type, *Group C* IDH wild-type-TERT wild-type, *Group D* IDH mutated-TERT mutated

^b^One-way ANOVA was applied for age, and Pearson’s chi-square test was done for others in statistical analysis

**Fig. 3:**
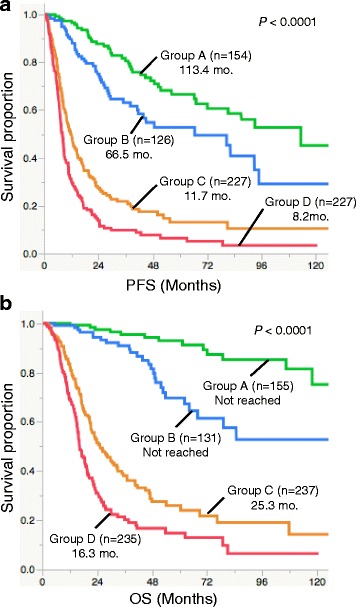
Kaplan-Meier analysis of progression free survival (PFS) and overall survival (OS) of molecular groups in Cohort 1. **a**. PFS of each molecular group (*n* = 734). Median PFS was 113.4 months for Group A, 66.5 months for Group B, 11.7 months for Group C, and 8.2 months for Group D (*P* < 0.0001, Log-rank test). Notably, Group D showed significantly shorter PFS than other groups (*P* < 0.0001, Log-rank test). **b**. OS of each molecular group (*n* = 758). Median OS was not reached for Groups A and B, 25.3 months for Group C, and 16.3 months for Group D (*P* < 0.0001, Log-rank test). Group D showed significantly shorter survival than any other groups (*P* < 0.0001, Log-rank test). Group A, *IDH* mutated- *TERT* mutated; Group B, *IDH* mutated-*TERT* wild-type; Group C, *IDH* wild-type-*TERT* wild-type; Group D, *IDH* mutated-*TERT* mutated

There was a significant difference in overall survival (OS) and progression-free survival (PFS) between each group (*p* < 0.0001, Log-rank test; Fig. 3). Group A showed the most favorable prognosis (median OS, not reached; median PFS, 113.4 months), followed by group B (median OS, not reached; median PFS, 66.5 months). Group D showed significantly shorter OS or PFS than any other group. Multivariate analysis using Cox regression models including clinicopathological information also revealed the significant prognostic impact of molecular classification, histological diagnosis, age, sex, and surgery (Table 3).

**Table 3:** Univariate and Multivariate Cox regression analysis for all cases of Cohort 1 (*n* = 758)

		Univariate						Multivariate					
		OS			PFS			OS			PFS		
		HR	95 % C.I.	p-value	HR	95 % C.I.	p-value	HR	95 % C.I.	p-value	HR	95 % C.I.	p-value
Histology	Astro grade II	0.91	0.518–1.594	0.74	1.23	0.788–1.906	0.37	0.58	0.323–1.027	0.06	0.92	0.585–1.461	0.74
	Astro grade III	2.14	1.410–3.245	0.0003	1.85	1.291–2.638	0.0008	1.02	0.658–1.566	0.95	0.99	0.682–1.447	0.97
	GBM	4.02	2.860–5.635	<0.0001	3.73	2.801–4.974	<0.0001	1.24	0.842–1.813	0.28	1.43	1.024–1.989	0.04
	Oligo grade II	0.25	0.127–0.504	<0.0001	0.48	0.313–0.749	0.001	0.42	0.207–0.846	0.02	0.6	0.381–0.928	0.02
	Oligo grade III	Ref			Ref			Ref			Ref		
Molecular group*	Group A	0.04	0.023–0.073	<0.0001	0.1	0.070–0.142	<0.0001	0.09	0.048–0.180	<0.0001	0.19	0.124–0.296	<0.0001
	Group B	0.12	0.079–0.184	<0.0001	0.18	0.126–0.242	<0.0001	0.29	0.172–0.480	<0.0001	0.33	0.218–0.492	<0.0001
	Group C	0.58	0.454–0.735	<0.0001	0.62	0.499–0.773	<0.0001	0.71	0.545–0.919	0.01	0.7	0.553–0.888	0.003
	Group D	Ref			Ref			Ref			Ref		
Location	Cerebrum	0.79	0.482–1.305	0.36	0.65	0.430–0.985	0.04	excluded by the factor selection with step-wise method			0.67	0.427–1.045	0.08
	Cerebrum with frontal Involvement	0.40	0.242–0.670	0.0005	0.31	0.203–0.475	<0.0001				0.5	0.316–0.779	0.002
	Other	Ref			Ref						Ref		
Age		1.048^a^	1.040–1.057	<0.0001	1.033^a^	1.027–1.040	<0.0001	1.02^a^	1.011–1.029	<0.0001	1.01^a^	1.003–1.018	0.007
Sex	M	Ref			Ref			Ref			Ref		
	F	0.91	0.724–1.134	0.39	0.95	0.781–1.150	0.58	0.72	0.572–0.905	0.005	0.81	0.663–0.982	0.03
KPS		0.971^b^	0.965–0.976	<0.0001	0.98^b^	0.974–0.985	<0.0001	0.99^b^	0.983–0.997	0.008	excluded by factor selection with step-wise method		
Surgery	Biopsy	1.85	1.416–2.427	<0.0001	1.41	1.092–1.817	0.008	2.05	1.522–2.755	<0.0001	1.6	1.204–2.121	0.001
	Removal	Ref			Ref			Ref			Ref		
Adjuvant Therapy	CRT	2.02	1.132–3.601	0.02	1.78	1.110–2.868	0.02	excluded by factor selection with step-wise method			excluded by factor selection with step-wise method		
	Chemo	0.84	0.392–1.792	0.65	0.87	0.472–1.587	0.64						
	RT	Ref			Ref								
	None	0.45	0.197–1.010	0.05	0.74	0.411–1.333	0.32						

*Astro grade II* diffuse astrocytoma, *Astro grade III* anaplastic astrocytoma, *Chemo* Chemotherapy, *C.I*. Coefficient interval, *CRT* chemoradiotherapy, *F* Female, *GBM* glioblastoma, *HR* hazard ratio, *KPS* Karnofsky Performance status, *M* Male, *mut* mutatant, *Oligo grade II* oligodendroglioma and oligoastrocytoma, *Oligo grade III* anaplastic oligodendroglioma and anaplastic oligoastrocytoma, *OS* overall survival, *PFS* progression free survival, *Ref* Reference, *RT* radiation therapy, *wt* wild type

^*^ *Group A* IDH mutated-TERT mutated, *Group B* IDH mutated-TERT wid-type, *Group C* IDH wild-type-TERT wild-type, *Group D* IDH mutated-TERT mutated

^a^ *HR* is for each one year increase, ^b^ *HR* is for each 1 % increase

The molecular groups were also associated with patient age, spatial distribution, and other specific genetic profiles. The *IDH*-mutated groups (Groups A and B) comprised younger patients than the *IDH* wild-type groups (Groups C and D; 43.3 vs. 60.0 years, respectively; *p* < 0.0001, Student’s t-test). Among the *IDH*-wild-type groups, Group C patients were younger than those in Group D (56.7 vs. 63.4 years, respectively; *p* < 0.0001, Student’s t-test). Tumors in the *IDH*-mutant groups commonly involved the frontal lobe. Thalamic or infratentorial tumors were the most common in Group C (72 and 85 %, respectively). The great majority of the Group A tumors showed 1p19q codeletion (93 %). Among the *IDH*-wild-type tumors, mutations in *H3F3A* or *HIST1H3B* (“histone H3 mutations”) were mostly observed in Group C. Deletion of *CDKN2A* was predominantly observed in *IDH*-wild-type groups (Groups C and D). The frequency of *TERT* mutation-type (C250T or C228T) did not differ between Group A and Group D (*p* = 0.11, chi-square test).

#### Multivariate Cox regression models revealed differences in prognostic impact of WHO grade among molecular groups

We performed Cox regression analysis in each group to investigate whether the molecular groups are prognostic markers independent of the histological diagnosis. In Group A, only the patients’ clinical backgrounds (age, KPS, and surgical history) were associated with overall survival after multivariate analysis, suggesting that this group was homogeneous regardless of the WHO grade (Additional file 2: Table S3). The 1p19q status was not significantly associated with survival by univariate Cox regression analysis, although the number of 1p19q intact tumors (*n* = 11/155) was too small for statistically conclusive results (Additional file 2: Table S3; Additional file 3: Figure S2). On the other hand, the WHO grade (II or III) was associated with OS and PFS in both univariate and multivariate analysis for Group B (Additional file 2: Table S4). In Group C, the WHO grade was significantly associated with OS and PFS in univariate analysis, whereas it was not prognostic in multivariate analysis (Additional file 2: Table S5). The WHO grade did not have significant impact on OS in both univariate and multivariate analysis for Group D (Additional file 2: Table S6). Collectively, the impact of WHO grade differed according to the molecular groups.

#### *Interaction between* TERT *and* MGMT *in* IDH*-wild-type GBM*

To investigate the impact of the *TERT* mutations and *MGMT* methylation in *IDH*-wild-type GBMs, we selected *IDH*-wild-type GBM cases from Cohort 1 who received concurrent temozolomide and radiation therapy with a dose of 50 – 65 Gy, and whose clinical information and *MGMT* status were available. In total, 260 GBM cases met these criteria (“Cohort 1 GBM”) and were further analyzed (Fig. 1).

In univariate analysis of Cohort 1 GBM, the *TERT* mutation status was not associated with either OS or PFS, whereas the *MGMT* methylation status was strongly associated with longer survival (Additional file 2: Table S7a). However, multivariate analysis revealed that *TERT* mutation was associated with OS and PFS. The discordance in these results suggested that the prognostic impact of *TERT* mutation may be affected by other factors. We next performed a multivariate Cox regression analysis incorporating *TERT* and *MGMT* interaction. This analysis revealed a significant interaction between *TERT* and *MGMT* for both OS and PFS (*P* = 0.0002 and 0.0342, respectively; Additional file 2: Table S7b). When compared with the *TERT* mutated-*MGMT* unmethylated group, the hazard ratio (HR) for OS incorporating the interaction was the lowest in the *TERT* mutated/*MGMT* methylated group (HR, 0.186), then the *TERT* wild-type/*MGMT* methylated group (HR, 0.392), finally the *TERT* wild-type/*MGMT* unmethylated group (HR, 0.476).

**Fig. 4:**
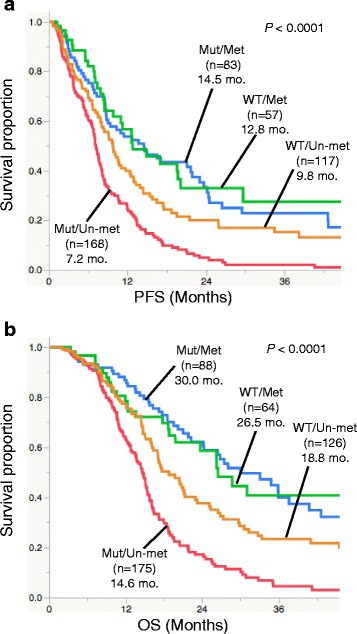
Kaplan-Meier analysis for survival, stratified by *TERT* and *MGMT* statuses in 453 GBM cases treated with radiation plus temozolomide. **a**. PFS of GBM cases (see text for definition). Median PFS was 14.5 months for *TERT* mutated-*MGMT* methylated (Mut/Met), 12.8 months for *TERT* wild-type-*MGMT* methylated (WT/Met), 9.8 months for *TERT* wild-type-*MGMT* unmethylated (WT/Un-met), and 7.2 months for *TERT* mutated-*MGMT* unmethylated (Mut/Un-met) (*P* < 0.0001, Log-rank test). The Mut/Un-met group showed shorter PFS than WT/Un-met (*P* = 0.0003, Log-rank test), whereas the differences in PFS between the *MGMT* methylated groups (between Mut/Met and WT/Met) was not significant (*P* = 0.62, Log-rank test). **b**. OS of the GBM cases. Median OS was 30.0 months for Mut/Met, 26.5 months for WT/Met, 18.8 months for WT/Un-met, and 14.6 months for Mut/Un-met (*P* < 0.0001, Log-rank test). The Mut/Un-met group had shorter OS than WT/Un-met (*P* < 0.0001, Log-rank test), whereas the difference in OS between Mut/Met and WT/Met was not significant (*P* = 0.83, Log-rank test). *GBM*, glioblastoma; *OS*, overall survival; *PFS*, progression free survival

To validate the findings in a larger series, we collected an independent cohort of 193 GBM cases as Cohort 2 (Fig. 1). This cohort was selected using the same criteria as the Cohort 1 GBMs (see above and Fig. 1), except that local histological diagnosis was employed. The frequency of *TERT* promoter mutations in Cohort 2 (60 %) was comparable to the GBMs from Cohort 1. The Cohort 1 and Cohort 2 GBMs were then combined and analyzed (Table 4). When stratified by *TERT* and *MGMT* statuses, their association with OS and PFS were as follows: 1) In the patients with *MGMT* methylated tumors, *TERT* status was not associated with either OS or PFS (*P* > 0.05, Log-rank test) 2) In the patients with *MGMT* unmethylated tumors, those with *TERT* mutant tumors showed shorter OS and PFS than those with *TERT* wild-type tumors (*P* < 0.05, Log-rank test); 3) lack of *MGMT* promoter methylation was associated with shorter OS and PFS in both *TERT* wild-type (Group C GBM) and *TERT* mutant (Group D GBM) groups (*P* < 0.05, Log-rank test; Fig. 4). As a result, patients with the *TERT* mutated-*MGMT* unmethylated GBMs had the shortest survival (median OS, 14.6 months), whereas those with *TERT* mutated-*MGMT* methylated GBMs survived the longest (median OS, 30.0 months).

**Table 4:** Patient background and molecular status of analysis for GBM cohort

Cohort	All^a^	Cohort 1	Cohort 2	*P*-value^b^
Total	453	260	193	
Mean Age at diagnosis	61.0	59.3	63.3	0.0007
Sex M/F	249/204	152/108	97/96	0.0827
Location^c^				
Cereberal with frontal involvement	152 (34)	87 (33)	65 (34)	0.985
Cerebral (other)	277 (61)	160 (62)	117 (61)	
Other	22 (5)	13 (5)	9 (5)	
KPS				
100	56 (12)	12 (5)	44 (23)	<0.0001
90	132 (29)	81 (31)	51 (26)	
80	111 (25)	73 (28)	38 (20)	
70	85 (19)	53 (20)	32 (17)	
− 60	69 (15)	41 (16)	28 (15)	
*TERT/MGMT*				
Mut/Met	88 (19)	48 (18)	40 (21)	0.921
Mut/Un-met	175 (39)	100 (38)	75 (39)	
Wt/Met	64 (14)	38 (15)	26 (13)	
Wt/Un-met	126 (28)	74 (28)	52 (27)	
Surgery				
Removal	421 (93)	238 (92)	183 (95)	
Biopsy only	32 (7)	22 (8)	10 (5)	0.178

^a^IDH1/2 mutated cases were excluded for this analysis (see Materials and methods in the manuscript)

^b^Student’s t-test was applied for the statistical analysis of age, and Pearson’s chi-square test was done for others

^c^Data of Location was not available in 2 cases of Cohort2

*F* Female, *GBM* glioblastoma, *KPS* Karnofsky Performance Status, *M* Male, *Met* MGMT methylated, *Mut* TERT mutated, *N/A* not available, *Un-met* MGMT unmethylated, *wt* TERT wild-type

A multivariate Cox regression model incorporating age, gender, cohort, KPS, tumor location, surgical history, *TERT*, and *MGMT* revealed that the interaction between *TERT* and *MGMT* was significant for OS in the combined GBM cohort of 453 cases. Against a reference of the *TERT* mutant-*MGMT* unmethylated GBMs, the HR for OS incorporating the interaction was the lowest in the *TERT* mutant-*MGMT* methylated GBM (HR, 0.266), followed by the *TERT* wild-type-*MGMT* methylated (HR, 0.317), and the *TERT* wild-type-*MGMT* unmethylated GBMs (HR, 0.542). For PFS, *TERT* and *MGMT* independently influenced survival (Table 5).

**Table 5:** Univariate and multivariate Cox regression analysis for GBM cohort (*n* = 453)

		Univariate						Multivariate					
		OS			PFS			OS			PFS		
		HR	95 % C.I.	p-value	HR	95 % C.I.	p-value	HR	95 % C.I.	p-value	HR	95 % C.I.	p-value
Cohort	Cohort 1	0.81	0.643–1.030	0.09	1.11	0.884–1.398	0.37	excluded by factor selection with step-wise method			excluded by factor selection with step-wise method		
	Cohort 2	Ref			Ref								
Location	Cerebrum	0.91	0.504–1.624	0.74	0.81	0.489–1.357	0.43	excluded by factor selection with step-wise method			excluded by factor selection with step-wise method		
	Cerebrum with Frontal Involvement	0.93	0.511–1.705	0.82	0.78	0.458–1.317	0.35						
	Other	Ref			Ref								
Age		1.015^a^	1.004–1.025	0.006	1.004^a^	0.995–1.013	0.38	1.01^a^	1.004–1.025	0.008	excluded by factor selection with step-wise method		
Sex	M	Ref			Ref			excluded by factor selection with step-wise method			excluded by factor selection with step-wise method		
	F	0.72	0.570–0.918	0.008	0.78	0.621–0.975	0.03						
KPS		0.992^b^	0.985–0.999	0.03	0.999^b^	0.992–1.006	0.74	0.99^b^	0.982–0.997	0.009	excluded by factor selection with step-wise method		
Surgery	Biopsy	2.59	1.725–3.899	<0.0001	2.06	1.349–3.130	0.0008	3.15	2.082–4.769	<0.0001	2.22	1.454–3.380	0.0002
	Removal	Ref			Ref			Ref			Ref		
*TERT*	wild-type	0.73	0.568–0.925	0.01	0.73	0.581–0.922	0.008	Interacts with MGMT		<0.0001	0.65	0.513–0.821	0.0003
	mutated	Ref			Ref						Ref		
*MGMT*	Methylated	0.43	0.325–0.561	<0.0001	0.51	0.396–0.656	<0.0001	Interacts with TERT		<0.0001	0.48	0.368–0.613	<0.0001
	Unmethylated	Ref			Ref						Ref		
*TERT-MGMT*	TERT-MGMT interaction									0.006	excluded by factor selection with step-wise method		
	Wt/Met							0.32					
	Mut/Met							0.27					
	Wt/Un-met							0.54					
	Mut/Un-met							Ref					

*Chemo* Chemotherapy, *C.I.* Coefficient interval, *CRT* chemoradiotherapy, *F* Female, *GBM* glioblastoma, *HR* hazard ratio, *KPS* Karnofsky Performance status, *M* Male, *Met* MGMT methylated, *Mut* TERT mutated, *OS* overall survival, *PFS* progression free survival, *Ref* Reference, *RT* radiation therapy, *Un-met* MGMT unmethylated, *Wt* TERT wild-type

^a^HR is for each one year increase

^b^HR is for each 1 % increase

When Cohort 2 was analyzed separately, univariate and multivariate Cox regression analysis for OS and PFS showed that the *TERT* and *MGMT* statuses were independent prognostic factors. Interaction between *TERT* and *MGMT* was not statistically significant in this cohort (Additional file 2: Table S8).

## Discussion and conclusions

In this study, we investigated the efficacy of *TERT* promoter mutation as a diagnostic and/or prognostic marker in combination with *IDH* mutation and *MGMT* methylation in a large series of newly diagnosed adult gliomas with detailed clinical information and a relatively homogeneous background, including postoperative treatment, in particular for GBM. Our results indicated that molecular classification based on the *IDH* and *TERT* statuses defines four groups within adult diffuse gliomas of grade II-IV, each showing distinct clinical-pathological features such as histological type, age, or tumor location. *TERT* promoter mutation was a favorable prognostic factor in *IDH* mutated tumors, whereas it was an unfavorable prognostic factor in *IDH* wild-type tumors. The most striking finding was that the prognostic impact of *TERT* promoter mutation may depend not only on the *IDH* status but also on the *MGMT* methylation status.

The prognostic value of *TERT* mutation in GBMs has been controversial. Some have suggested that *TERT* status did not have an impact on OS in *IDH* wild-type GBMs after adjusting by age and gender [6, 26], whereas others found an adverse prognostic impact of *TERT* mutation by multivariate analysis including treatment [16, 30]. This discrepancy may be due to insufficient cohort size or uneven treatment in some of the cohorts. Clinical background may also be a confounding factor. For example, *TERT* mutation is strongly associated with higher age, which in itself is a well-known prognostic factor and affects treatment choice. In the present study, the prognostic impact of *TERT* was validated in two independent cohorts of GBMs with similar clinical backgrounds in which molecular tests were thoroughly performed.

A potentially more significant possibility is the presence of additional confounding factors that influence survival of GBM patients in association with the *TERT* status. *MGMT* methylation is a well-established favorable prognostic marker for survival in GBM patients, and predicts the response to temozolomide in elderly GBM patients [9, 22, 34]. We therefore investigated the potential interaction between *TERT* mutation and *MGMT* methylation status in newly diagnosed GBM patients who received a standard treatment with concomitant temozolomide and radiation therapy in Cohort 1 as well as the combined GBM cohort. Our results indicate that the prognostic impact of *TERT* mutation is strongly influenced by the *MGMT* methylation status. The limitation of this study is that the interaction was validated in the combined cohort but not in the Cohort 2 alone, most likely because of the insufficient cohort size. A further validation in a larger cohort is warranted.

Our finding that the subset of GBMs defined as having *TERT* mutation and unmethylated *MGMT* have the poorest prognosis has important clinical implications. Our results conclusively demonstrated that *TERT* mutation is one of the most powerful predictors of survival in GBM patients, along with the *MGMT* methylation status. It has been shown that temozolomide is effective for GBM with methylated *MGMT*. Patients with *MGMT* unmethylated GBM who receive only minimal benefit from current standard treatments including temozolomide are the primary population who require new therapeutic agents [10]. *TERT* may thus serve as an alternative therapeutic target for these patients.

Currently, Imetelstat is the only telomerase inhibitor that has been tested in clinical trials [23]. Imetelstat is an oligonucleotide inhibitor to *TERC*, an RNA subunit of telomerase, but not a direct inhibitor of TERT. Clinical anti-oncogenic activity of Imetelstat has yet to be demonstrated. In addition to its activity as the reverse transcriptase for telomerase, TERT is reported to have activity as an RNA-dependent RNA polymerase (RdRP) [20]. RdRP plays an essential role in RNA silencing by generating double-stranded RNAs, which are processed into microRNAs [20, 21]. It has been suggested that TERT is involved in diverse cellular functions, such as heterochromatin formation or maintenance of tumor-initiating cells through its RdRP activity. Recently, a specific inhibitor for RdRP that suppresses growth of platinum-resistant ovarian cancer cell lines with *TERT* mutation and upregulation has been proposed [35]. A pre-clinical study in GBM is underway (Takahashi, in preparation).

The biological mechanism for the interaction between *TERT* mutation and *MGMT* methylation that influences patient survival is currently unclear. A broad spectrum of the biological consequences of *TERT* activation, for example by microRNAs generated through TERT-RdRP, may affect the response to chemotherapy and/or radiotherapy.

One of the major challenges of the integrated diagnosis system for adult gliomas is whether histological diagnosis or WHO grading still have survival impact after stratification by molecular information. In our study, a multivariate analysis revealed that the histological diagnosis continues to be a significant predictor of survival. We further investigated the prognostic value of histological diagnosis in relation to clinical information in each subgroup.

We found that the WHO grade had no significant impact on OS in either Groups A or D, suggesting that each of these groups may be regarded as clinically homogeneous (Additional file 2: Table S10). Virtually all tumors allocated to Group A are molecularly scored as oligodendroglial tumors, because the great majority of them presented total 1p19q codeletion. There remains a controversy whether or not *TERT*-*IDH* co-mutated, but not 1p19q codeleted, tumors are oligodendrogliomas. Along with a large-scale analysis reported by Eckel-Passow et al. [6], those paradoxical cases showed prognosis comparable to the codeleted tumors, suggesting that those tumors could be regarded as oligodendrogliomas. Group D tumors showed universally dismal prognosis regardless of the histological subtype, indicating that this group is biologically *bona fide* GBM and that grade II-III tumors in this group should be regarded as under-diagnosed GBM.

On the other hand, a univariate analysis identified WHO grade as a factor significantly associated with survival in Group B (*IDH* mutated-*TERT* wild-type, representing astrocytomas), and a multivariate analysis for survival in Group B also identified WHO grade (II or III) as a prognostic factor in the present study. These results are in contrast to recent reports that found no survival impact of WHO grade in *IDH* mutated astrocytomas [27, 29]; however, it supports the results of another study [6]. So far, there are no molecular markers that define WHO grades. Our results and others, however, suggest that WHO grade may still be relevant in some types of gliomas. The limitation of these studies is that the treatment of low-grade astrocytomas may be inconsistent and have potentially confounded the results. A prospective study with homogeneously treated patients in clinical trials would be needed to clarify the significance of WHO grade in the era of molecular diagnostics.

The Group C remains enigmatic. This triple negative group has highly heterogeneous backgrounds, including histological diagnosis, tumor location, and other genetic traits. This group mainly consists of adult GBMs, however it also contains pediatric types of GBM that harbor *H3F3A* mutations and indolent tumors resembling pediatric lower grade gliomas with *BRAF* mutations. A large scale analysis based on genome-wide methylation analysis identified tumors exhibiting methylation profiles similar to pilocytic astrocytomas, as well as tumors resembling pediatric GBMs in the *IDH* wild-type group [5]. Although WHO grade II and III tumors in Group C showed slightly better outcomes compared with the grade IV counterparts, the prognoses of the grade IV tumors in Group C were comparable with Group D. This shows that at least histologically proven GBMs in Group C are clinically relevant GBMs. The question remains whether the triple negative grade II-III tumors are biologically under-diagnosed GBMs, or if at least some of them form a separate sub-entity of genuine “Diffuse (Anaplastic) astrocytoma, *IDH* wild-type” tumors with an intermediate prognosis between those with *IDH* mutations and GBM. Further studies to establish molecular markers that unequivocally define GBM (e.g., EGFR amplification, monosomy 10/trisomy 7, or co-gain of chromosomes 19/20 [7, 28]) on a larger collection of the triple negative/Group C tumors are warranted.

In this study, using a large cohort of newly diagnosed adult gliomas with precise clinical information, we demonstrated that molecular classification using *IDH* and *TERT* statuses is a strong prognostic marker of adult gliomas. The *IDH*-*TERT* classification efficiently identifies molecularly defined oligodendrogliomas and astrocytomas equivalent to the *IDH*-1p/19q-based classification. Although an accurate determination of total 1p/19q codeletion may require laborious and expensive molecular tests, examination of two hotspots in the *TERT* promoter is comparatively simple. Moreover, we found that a combination of *TERT* mutation and the *MGMT* methylation status classified GBMs into clinically relevant subgroups, identifying *TERT* mutated-*MGMT* unmethylated tumors as having the most severe outcome, and thus highlighting TERT as a primary target for novel therapies. Thus, by using *TERT* mutation as an additional biomarker, the molecular classification presented in this study will refine the integrated diagnostic system and prognostication of glioma patients. Our results emphasize the importance of combining molecular markers such as *IDH*, 1p/19q, *TERT*, and *MGMT* for accurate molecular diagnosis, prognostication, and the choice of treatment in clinical trials, as well as in routine clinical practice for glioma patients.

## Additional files

Additional file 1: Supplementary Information. (DOCX 141 kb) Additional file 2: Table S1. Molecular and clinical characteristics of Cohort 1 (*n* = 758). **Table S2.** Molecular and clinical characteristics of GBM cohort (*n* = 453). **Table S3.** Univariate and multivariate Cox regression analyses for Group A (*IDH* mutated-*TERT* mutated) tumors in Cohort 1 (*n* = 155). **Table S4.** Univariate and multivariate Cox regression analyses for Group B (*IDH* mutated-*TERT* wild-type) tumors in Cohort 1 (*n* = 131). **Table S5.** Univariate and multivariate Cox regression analyses for Group C (*IDH* wild-type-*TERT* wild-type) tumors in Cohort 1 (*n* = 237). **Table S6.** Univariate and multivariate Cox regression analyses for Group D (*IDH* wild-type-*TERT* mutated) tumors in Cohort 1 (*n* = 235). **Table S7.** Univariate and multivariate Cox regression analyses for GBM in Cohort 1 (*n* = 260). **Table S8.** Univariate and multivariate Cox regression analyses for GBM in Cohort 2 (*n* = 193). **Table S9.** Background of combined GBM cohort stratified by *TERT* and *MGMT* status (*n* = 453). **Table S10.** Survival time and WHO grade in each molecular subgroup of Cohort 1 (*n* = 758). (XLSX 254 kb) Additional file 3: Figure S1. Distributions of molecular alterations according to histology in Cohort 1. **Figure S2.** Kaplan-Meier analysis for Group A cases stratified by 1p/19q status. **Figure S3.** Kaplan-Meier analyses for GBM cases in Cohorts 1 and 2. (PPTX 172 kb)
