# Supplementary material for: A combination of TERT promoter mutation and MGMT methylation status predicts clinically relevant subgroups of newly diagnosed glioblastomas
Source: Acta Neuropathol Commun. 2016 Aug 8;4:79. doi: 10.1186/s40478-016-0351-2 (PMC4977715; doi:10.1186/s40478-016-0351-2)
Supplement: Additional file 1: — Supplementary Information. (DOCX 141 kb) [file 40478_2016_351_MOESM1_ESM.docx]

**Supplementary Information**

**A combination of TERT promoter mutation and MGMT methylation status predicts clinically relevant subgroups of newly diagnosed glioblastomas**

Authors list

Hideyuki Arita, Kai Yamasaki, Yuko Matsushita, Taishi Nakamura, Asanao Shimokawa, Hirokazu Takami, Shota Tanaka, Akitake Mukasa, Mitsuaki Shirahata, Saki Shimizu, Kaori Suzuki, Kuniaki Saito, Keiichi Kobayashi, Fumi Higuchi, Takeo Uzuka, Ryohei Otani, Kaoru Tamura, Kazutaka Sumita, Makoto Ohno, Yasuji Miyakita, Naoki Kagawa^,^, Naoya Hashimoto, Ryusuke Hatae, Koji Yoshimoto, Naoki Shinojima, Hideo Nakamura, Yonehiro Kanemura, Yoshiko Okita, Manabu Kinoshita, Kenichi Ishibashi, Tomoko Shofuda, Yoshinori Kodama, Kanji Mori, Yusuke Tomogane, Junya Fukai, Koji Fujita, Yuzo Terakawa, Naohiro Tsuyuguchi, Shusuke Moriuchi, Masahiro Nonaka, Hiroyoshi Suzuki, Makoto Shibuya, Taketoshi Maehara, Nobuhito Saito, Motoo Nagane, Nobutaka Kawahara, Keisuke Ueki, Toshiki Yoshimine, Etsuo Miyaoka, Ryo Nishikawa, Takashi Komori, Yoshitaka Narita, Koichi Ichimura

# Supplementary Tables

**Supplementary Tables S1-9** are provided in the attached Excel file.

## Supplementary Table S1. Molecular and clinical characteristics of Cohort 1 (n = 758)

## Supplementary Table S2. Molecular and clinical characteristics of GBM cohort (n = 453)

## Supplementary Table S3.

### 3a. Univariate Cox regression analysis for Group A (*IDH* mutated-*TERT* mutated) tumors in Cohort 1 (n = 155).

### 3b. Multivariate Cox regression analysis for Group A (*IDH* mutated-*TERT* mutated) tumors in Cohort 1 (n = 146).

## Supplementary Table S4

### 4a. Univariate Cox regression analysis for Group B (*IDH* mutated-*TERT* wild-type) tumors in Cohort 1 (n = 131).

### 4b. Multivariate Cox regression analysis for Group B (*IDH* mutated-*TERT* wild-type) tumors in Cohort 1 (n = 124).

## Supplementary Table S5

### 5a. Univariate Cox regression analysis for Group C (*IDH* wild-type-*TERT* wild-type) tumors in Cohort 1 (n = 237).

### 5b. Multivariate Cox regression analysis for Group C (*IDH* wild-type-*TERT* wild-type) tumors in Cohort 1 after selection by step-wise method (n = 237).

## Supplementary Table S6

### 6a. Univariate Cox regression analysis for Group D (*IDH* wild-type-*TERT* mutated) tumors in Cohort 1 (n = 235).

### 6b. Multivariate Cox regression analysis for Group D (*IDH* wild-type-*TERT* mutated) tumors in Cohort 1 after selection by step-wise method (n = 235).

## Supplementary Table S7

### 7a. Univariate Cox regression analysis for GBM in Cohort 1 (n = 260).

### 7b. Multivariate Cox regression analysis for GBM in Cohort 1 after selection by step-wise method (n = 260).

## Supplementary Table S8

### 8a. Univariate Cox regression analysis for GBM in Cohort 2 (n = 193).

### 8b. Multivariate Cox regression analysis for GBM in Cohort 2 after selection by step-wise method (n = 193).

## Supplementary Table S9. Background of combined GBM cohort stratified by *TERT* and *MGMT* status (n = 453)

## Supplementary Table S10. Survival time and WHO grade in each molecular subgroup of Cohort 1 (n = 758)

# Supplementary Methods

## Outline of Molecular analysis

Almost all molecular analyses were performed in two laboratories: the National Cancer Center Research Institute (NCC), Tokyo, Japan, and the Osaka National Hospital (ONH), Osaka, Japan. Target sequencing for *IDH1/2*, *TERT* promoter, *BRAF*, and *H3F3A* hotspots was performed and assessed in either lab. All pyrosequencing analyses for *MGMT* promoter methylation status were performed at NCC. Multiplex ligation-dependent probe amplification (MLPA) for 1p/19q status was conducted in either lab, and data were analyzed in a unified workflow at NCC as described below. In the Dokkyo University and Tokyo University cohort, 1p19q codeletion was assessed by microsatellite analysis, as previously reported [8].

## Sanger sequence

In ONH, genomic regions of interest were amplified by PCR with gene-specific primers as previously described [1, 3, 9] (also listed in **Supplementary Table S10**), and TaKaRa Ex Taq® (TAKARA BIO INC., Shiga, Japan) using an Applied Biosystems GeneAmp PCR System 9700 (Thermo Fisher Scientific, Waltham, MA). PCR products were purified by a QIAquick Gel Extraction Kit (Qiagen, Valencia, CA), and then sequenced with sequencing primer (*IDH1*) or PCR forward primer (*IDH2*, *BRAF*, and *TERT*) and a BigDye® Terminator V1.1 Cycle Sequencing Kit (Thermo Fisher Scientific) using an ABI 3130xL Genetic Analyzer (Thermo Fisher Scientific). In NCC, target regions were amplified by gene specific primers (**Supplementary Table S11**) and an AmpliTaq Gold 360 (Thermo Fisher Scientific) using a TProfessional Thermocycler (Biometra, Göttingen, Germany). An ExoSAP-IT (Affymetrix Inc, Santa Clara, CA) was used for purification of PCR products. All purified products were sequenced by M13 primer and a BigDye® Terminator V3.1 Cycle Sequencing Kit using a ABI 3130xL Genetic Analyzer. The BigDye® Terminator V1.1 was used only for *TERT* promoter sequencing.

## Pyrosequencing

Templates for pyrosequencing were prepared by amplifying genomic DNA (10 ng) using one-sided biotinylated primer pairs. An AmpliTaq Gold 360 DNA polymerase (Thermo Fisher Scientific) was used for amplification of each template, except *H3F3A*. The Pyromark PCR Kit (Qiagen) was used for amplification of *H3F3A*. Single strand template preparation and purification were performed as manufacturer’s recommendations with 10 μl PCR products. Details of PCR and pyrosequencing primers are described in **Supplementary** **Table S12**. Pyrosequencing was performed using a Pyromark Gold Q96 Reagents and the PyroMark Q96 software (version 2.5.7) on a PSQ96 pyrosequencer (Qiagen). The concordance of results between Sanger sequencing and pyrosequencing was independently cross-validated in Kyusyu University using 73 glioma samples.

## *IDH1/2* hotspot mutations

*IDH1/2* status was assessed mainly by pyrosequencing and partly by Sanger sequencing, as previously reported [2]. Assays for *IDH1/2* pyrosequencing were designed for all possible mutations by the mutation specific pyrogram patterns, which have been shown to detect at least 5% of the *IDH1* R132H allele and 10% of the *IDH2* R172K allele.

## *TERT* promoter mutations

The hotspot mutations of the *TERT* promoter, termed C228T and C250T, were analyzed by Sanger sequencing and/or pyrosequencing as previously reported [1]. Because of the high GC content in the target region of amplification, 20% of 360 GC enhancer was added to the PCR reaction premix. The 3′ end of the pyrosequencing primer was designed immediately upstream of the C250 hotspot. Pyrosequencing of the reverse complement strand was performed concurrently to confirm the results.

## *BRAF* and *H3F3A* hotspot mutations

The presence of *BRAF* and *H3F3A* hotspot mutations was assessed in almost all cases with *IDH* wild type gliomas using Sanger sequencing or pyrosequencing. In ONH, *IDH* mutant gliomas were sequenced for *BRAF* and *H3F3A*.

## *MGMT* promoter methylation

In all samples, the methylation status of the *MGMT* promoter was analyzed using a customized pyrosequencing assay, essentially as previously described with some modifications [6]. Briefly, bisulfite modification of genomic DNA samples was performed using an EZ DNA Methylation^TM^ Kit (Zymo Research Corporation, Irvine, CA) as manufacturer’s recommendations. Templates for pyrosequencing were amplified by Pyromark PCR Kit (Qiagen). The average extent of methylation was calculated for the 16 CpG sites and used to represent the methylation level. A cut-off for methylation was determined to best predict outcome in an independent series of 276 newly diagnosed GBMs from patients treated with radiation and temozolomide. Accordingly, the cut-off was defined as 16% or above for methylation. The details of the pyrosequencing protocol for *MGMT* methylation will be described elsewhere (Ichimura, manuscript in preparation).

## 1p/19q co-deletion and *CDKN2A* deletion

A SALSA MLPA KIT probemix (P088-C1, MRC-Holland, Amsterdam, Netherlands) was used for copy number analysis of 1p/19q as previously described [7]. This kit contains 16 and 11 probes for 1p and 19q, respectively, as well as three probes for the *CDKN2A* gene. The results were analyzed using Coffalyser.NET (MLC-Holland) software. Intra-sample normalization was performed on the control probes. Single regression was performed for control and tumor data slope correction. The reported cutoff for deletion or gain of an individual probe is the corrected ratio < 0.8 or > 1.2, respectively [5]. The total 1p/19q codeletion was scored as having an average value for the ratio of both 1p and 19q probes as < 0.8, with no more than two probes exceeding 1.0 in each chromosomal arm. The details of copy number analysis of 1p/19q will be described elsewhere (Yamasaki, manuscript in preparation). The cut-off value of heterozygous or homozygous deletion of *CDKN2A* was defined as having an average ratio of the three probes between 0.4–0.7 or < 0.4, respectively.

## Array comparative genomic hybridization (aCGH)

aCGH was performed in selected tumors at NCC as described previously [1]. Briefly, 200 ng of tumor genomic DNA was labeled by Cy5-dUTP after digestion with restriction enzymes AluI and RsaI. Reference DNA (Promega, Madison, WI) was labeled with Cy3-dUTP. The labeled DNA was mixed with Cot-1 DNA (Agilent Technologies, Santa Clara, CA) and hybridized at 65°C for 24 h to SurePrint G3 Human CGH microarrays 8 × 60 K (Agilent Technologies). Agilent CytoGenomics software (Agilent Technologies) was used to analyze and visualize chromosomal gains and losses. The copy number status of the whole chromosomal arms was intuitively interpreted and converted into numeric data as described previously [4].

## Supplementary Table S10. Sequences of primers used in ONH for amplification and Sanger sequencing.

| gene | primer | sequence |
| --- | --- | --- |
| *IDH1* [3] | PCR-forward | 5′-AATGAGCTCTATATGCCATCACTG-3′ |
|  | PCR-reverse | 5′-TTCATACCTTGCTTAATGGGTGT-3′ |
|  | sequencing | 5′-GCCATCACTGCAGTTGTAGGTTA-3′ |
| *IDH2* [9] | PCR-forward | 5′-TTGTTGCTTGGGGTTCAAAT-3′ |
|  | PCR-reverse | 5′-TGTGGCCTTGTACTGCAGAG-3′ |
| *BRAF*-V600 | PCR-forward | 5′-CCTAACACATTTCAAGCCCCA-3′ |
|  | PCR-reverse | 5′-CACTGATTTTTGTGAATACTGGGA-3′ |
| *TERT* promoter [1] | PCR-forward | 5′-TCCCTCGGGTTACCCCACAG-3′ |
|  | PCR-reverse | 5′-AAAGGAAGGGGAGGGGCTG-3′ |

## Supplementary Table S11. Sequence of primers used in NCC for amplification and Sanger sequencing.

| ***IDH1* (for codon 132)** | |
| --- | --- |
| Forward primer | 5′-M13-TGAGCTCTATATGCCATCACTGC-3′ |
| Reverse primer | 5′-CAATTTCATACCTTGCTTAATGGG-3′ |
| ***IDH2* (for codon 172)** | |
| Forward primer | 5′-M13-TGGTCTGGCTGTGTTGTTGCTTG-3′ |
| Reverse primer | 5′-CAGAGACAAGAGGATGGCTAGG-3′ |
| ***TERT* promoter (for C250 and C228)** | |
| Forward primer | 5′-M13R-M13-CCAGCTCCGCCTCCTCCG-3′ |
| Reverse primer | 5′-GCTGCCTGAAACTCGCGCC-3′ |
| ***H3F3A* (for K27 and G34)** | |
| Forward primer | 5′-M13-GGTGATCGTGGCAGGAAA-3′ |
| Reverse primer | 5′-CCTCCAGGTAAGATTATGGCTTC-3′ |
| ***BRAF* (for codon 600)** | |
| Forward primer | 5′-M13-TTTGTGAATACTGGGAACTATGAAA-3′ |
| Reverse primer | 5′-TCATCCTAACACATTTCAAGCC-3′ |

## Supplementary Table S12. Sequences of primers used in NCC for amplification and pyrosequencing.

| ***IDH1* (for codon 132)** [2] | |
| --- | --- |
| Forward primer | 5′-CAAAAATATCCCCCGGCTTG-3′ |
| Reverse primer | 5′-bio-CAACATGACTTACTTGATCCCC-3′ |
| Sequence primer | 5′-ACCTATCATCATAGGT-3′ |
| Dispensation order | GATCATGTCATG |
| Sequence to analyze | CDTCATGCTTAT |
| ***IDH2* (for codon 172)** [2] | |
| Forward primer | 5′-ACATCCCACGCCTAGTCCC-3′ |
| Reverse primer | 5′-bio-TCTCCACCCTGGCCTACCTG |
| Sequence primer | 5′-CCCATCACCATTGGC-3′ |
| Dispensation order | GTACTGTCACGC |
| Sequence to analyze | ANGCACGCCCAT |
| ***IDH2* (for R172K confirmation)** | |
| Forward primer | 5′-ACATCCCACGCCTAGTCCC-3′ |
| Reverse primer | 5′-bio-TCTCCACCCTGGCCTACCTG-3′ |
| Sequence primer | 5′-CAAGCCCATCACCATTGGCA-3′ |
| Dispensation order | ACTGCACGC |
| Sequence to analyze | NGCACGCCCATGGCGACCAGGTA |
| ***TERT* promoter (for C250 and C228)** | |
| Forward primer | 5′-CCAGCTCCGCCTCCTCCG-3′ |
| Reverse primer | 5′-bio-GCTGCCTGAAACTCGCGCC-3′ |
| Sequence primer | 5′-ACCCCGCCCCGTCCCGACCCCT-3′ |
| Dispensation order | GGTCGGTCCGCAGCCTCG |
| Sequence to analyze | YCCGGGTCCCCGGCCCAGCCCCYTCCGGG |
| ***TERT* promoter (for C250 and C228) in reverse complement strand** | |
| Forward primer | 5′-GCTGCCTGAAACTCGCGCC-3′ |
| Reverse primer | 5′-bio-CCAGCTCCGCCTCCTCCG-3′ |
| Sequence primer | 5′-AGGGGCTGGGAGGGCCCGGA-3′ |
| Dispensation order | CAGGCTGCGG |
| Sequence to analyze | G/AGGGGCTGGGCCGGGGA |
| ***H3F3A* (for K27 and G34)** | |
| Forward primer | 5′-CATGGCTCGTACAAAGCAGA-3′ |
| Reverse primer | 5′-bio-CAAGAGAGACTTTGTCCCATTTTT-3′ |
| Sequence primer | 5′-GGCTACAAAAGCCGCTCGC-3′ |
| Dispensation order | ACTGGTGAGAC |
| Sequence to analyze | NGGGTGAAGAAACCTCATCGTTACAG |
| ***H3F3A* (for G34 confirmation)** | |
| Forward primer | 5′-CATGGCTCGTACAAAGCAGA-3′ |
| Reverse primer | 5′-bio-CAAGAGAGACTTTGTCCCATTTTT-3′ |
| Sequence primer | 5′-AGTGCGCCCTCTACTGGA-3′ |
| Dispensation order | ACTGGTGAGAC |
| Sequence to analyze | NGGGTGAAGAAACCTCATCGTTACAG |
| ***BRAF* (for V600 point mutation)** | |
| Forward primer | 5′-CTCTTCATAATGCTTGCTCTG-3′ |
| Reverse primer | 5′-bio-TAGTAACTCAGCAGCATCTCAG-3′ |
| Sequence primer | 5′-GGTGATTTTGGTCTAGCTAC-3′ |
| Dispensation order | GATCGATGATC |
| Sequence to analyze | A/TGT/AGAAATC |

# References

1 Arita H, Narita Y, Fukushima S, Tateishi K, Matsushita Y, Yoshida A, Miyakita Y, Ohno M, Collins VP, Kawahara N, Shibui S, Ichimura K. Upregulating mutations in the TERT promoter commonly occur in adult malignant gliomas and are strongly associated with total 1p19q loss. Acta Neuropathol. 2013;126:267-76.

2 Arita H, Narita Y, Matsushita Y, Fukushima S, Yoshida A, Takami H, Miyakita Y, Ohno M, Shibui S, Ichimura K. Development of a robust and sensitive pyrosequencing assay for the detection of IDH1/2 mutations in gliomas. Brain Tumor Pathol. 2015;32:22-30.

3 Bleeker FE, Lamba S, Leenstra S, Troost D, Hulsebos T, Vandertop WP, Frattini M, Molinari F, Knowles M, Cerrato A, Rodolfo M, Scarpa A, Felicioni L, Buttitta F, Malatesta S, Marchetti A, Bardelli A. IDH1 mutations at residue p.R132 (IDH1(R132)) occur frequently in high-grade gliomas but not in other solid tumors. Hum. Mutat. 2009;30:7-11.

4 Fukushima S, Otsuka A, Suzuki T, Yanagisawa T, Mishima K, Mukasa A, Saito N, Kumabe T, Kanamori M, Tominaga T, Narita Y, Shibui S, Kato M, Shibata T, Matsutani M, Nishikawa R, Ichimura K, Intracranial Germ Cell Tumor Genome Analysis C. Mutually exclusive mutations of KIT and RAS are associated with KIT mRNA expression and chromosomal instability in primary intracranial pure germinomas. Acta Neuropathol. 2014;127:911-25.

5 Jeuken J, Cornelissen S, Boots-Sprenger S, Gijsen S, Wesseling P. Multiplex ligation-dependent probe amplification: a diagnostic tool for simultaneous identification of different genetic markers in glial tumors. J. Mol. Diagn. 2006;8:433-43.

6 Mulholland S, Pearson DM, Hamoudi RA, Malley DS, Smith CM, Weaver JM, Jones DT, Kocialkowski S, Backlund LM, Collins VP, Ichimura K. MGMT CpG island is invariably methylated in adult astrocytic and oligodendroglial tumors with IDH1 or IDH2 mutations. Int. J. Cancer 2012;131:1104-13.

7 Okita Y, Narita Y, Miyakita Y, Ohno M, Matsushita Y, Fukushima S, Sumi M, Ichimura K, Kayama T, Shibui S. IDH1/2 mutation is a prognostic marker for survival and predicts response to chemotherapy for grade II gliomas concomitantly treated with radiation therapy. Int. J. Oncol. 2012;41:1325-36.

8 Ueki K, Nishikawa R, Nakazato Y, Hirose T, Hirato J, Funada N, Fujimaki T, Hojo S, Kubo O, Ide T, Usui M, Ochiai C, Ito S, Takahashi H, Mukasa A, Asai A, Kirino T. Correlation of histology and molecular genetic analysis of 1p, 19q, 10q, TP53, EGFR, CDK4, and CDKN2A in 91 astrocytic and oligodendroglial tumors. Clin. Cancer Res. 2002;8:196-201.

9 Yan H, Parsons DW, Jin G, McLendon R, Rasheed BA, Yuan W, Kos I, Batinic-Haberle I, Jones S, Riggins GJ, Friedman H, Friedman A, Reardon D, Herndon J, Kinzler KW, Velculescu VE, Vogelstein B, Bigner DD. IDH1 and IDH2 mutations in gliomas. N. Engl. J. Med. 2009;360:765-73.

# Supplementary Figure Legends

## Supplementary Figure S1. Distributions of molecular alterations according to histology in Cohort 1.

All 758 tumors in Cohort 1 are sorted according to histology. Each column represents one tumor. The mutation statuses of *IDH*, *TERT*, *H3F3A, and* *BRAF,* the copy number statuses of 1p/19q and *CDKN2A/B*, and *MGMT* methylation are shown. Centrally reviewed histology is indicated at the top. Gray or colored cells indicate absence or presence of alterations, respectively, blank cells denote lack of data. *Group A*, *IDH* mutated-*TERT* mutated; *Group B*, *IDH* mutated-*TERT* wild-type; *Group C*, *IDH* wild-type-*TERT* wild-type; *Group D*, *IDH* wild-type-*TERT* mutated. *AA*, anaplastic astrocytoma; *AO,* anaplastic oligodendroglioma; *AOA*, anaplastic oligoastrocytoma; *DA*, diffuse astrocytoma; *GBM*, glioblastoma; *OA*, oligoastrocytoma; *OL*, oligodendroglioma

## Supplementary Figure S2. Kaplan-Meier analysis for Group A cases stratified by 1p/19q status.

Progression free survival (PFS) (**a**) and overall survival (OS) (**b**), stratified by 1p/19q status in Group A. Neither PFS nor OS differed according to 1p/19q status (P > 0.05, Log-rank test).

## Supplementary Figure S3. Kaplan-Meier analyses for GBM cases in Cohorts 1 and 2.

**a**. Progression free survival (PFS) of Cohort 1 GBM cases (n = 255). Median PFS was 15.0 months for *TERT* mutated-*MGMT* methylated (Mut/Met), 11.2 months for *TERT* wild-type-*MGMT* methylated (WT/Met), 9.8 months for *TERT* wild-type-*MGMT* unmethylated (WT/Un-met), and 7.2 months for *TERT* mutated-*MGMT* unmethylated (Mut/Un-met) (P < 0.0001, Log-rank test). **b**. Overall survival (OS) of Cohort 1 GBM cases (n = 260). Median OS was 37.7 months for Mut/Met, 23.7 months for WT/Met, 20.4 months for WT/Un-met, and 15.2 months for Mut/Un-met (P < 0.0001, Log-rank test). **c**. PFS of Cohort 2 GBM cases (n = 170). Median PFS was 13.7 months for Mut/Met, 20.1 months for WT/Met, 12.5 months for WT/Un-met, and 7.1 months for Mut/Un-met (P = 0.0002, Log-rank test). **d**. OS of Cohort 2 GBM cases (n = 193). Median OS was 26.8 months for Mut/Met, 28.7 months for WT/Met, 18.8 months for WT/Un-met, and 13.6 months for Mut/Un-met (P < 0.0001, Log-rank test). *GBM*, glioblastoma; *OS*, overall survival; *PFS*, progression free survival.
