# Supplementary material for: A combination of TERT promoter mutation and MGMT methylation status predicts clinically relevant subgroups of newly diagnosed glioblastomas
Source: Acta Neuropathol Commun. 2016 Aug 8;4:79. doi: 10.1186/s40478-016-0351-2 (PMC4977715; doi:10.1186/s40478-016-0351-2)
Supplement: Additional file 3: Figure S1. — Distributions of molecular alterations according to histology in Cohort 1. Figure S2. Kaplan-Meier analysis for Group A cases stratified by 1p/19q status. Figure S3. Kaplan-Meier analyses for GBM cases in Cohorts 1 and 2. (PPTX 172 kb) [file 40478_2016_351_MOESM3_ESM.pptx]

## Slide 1
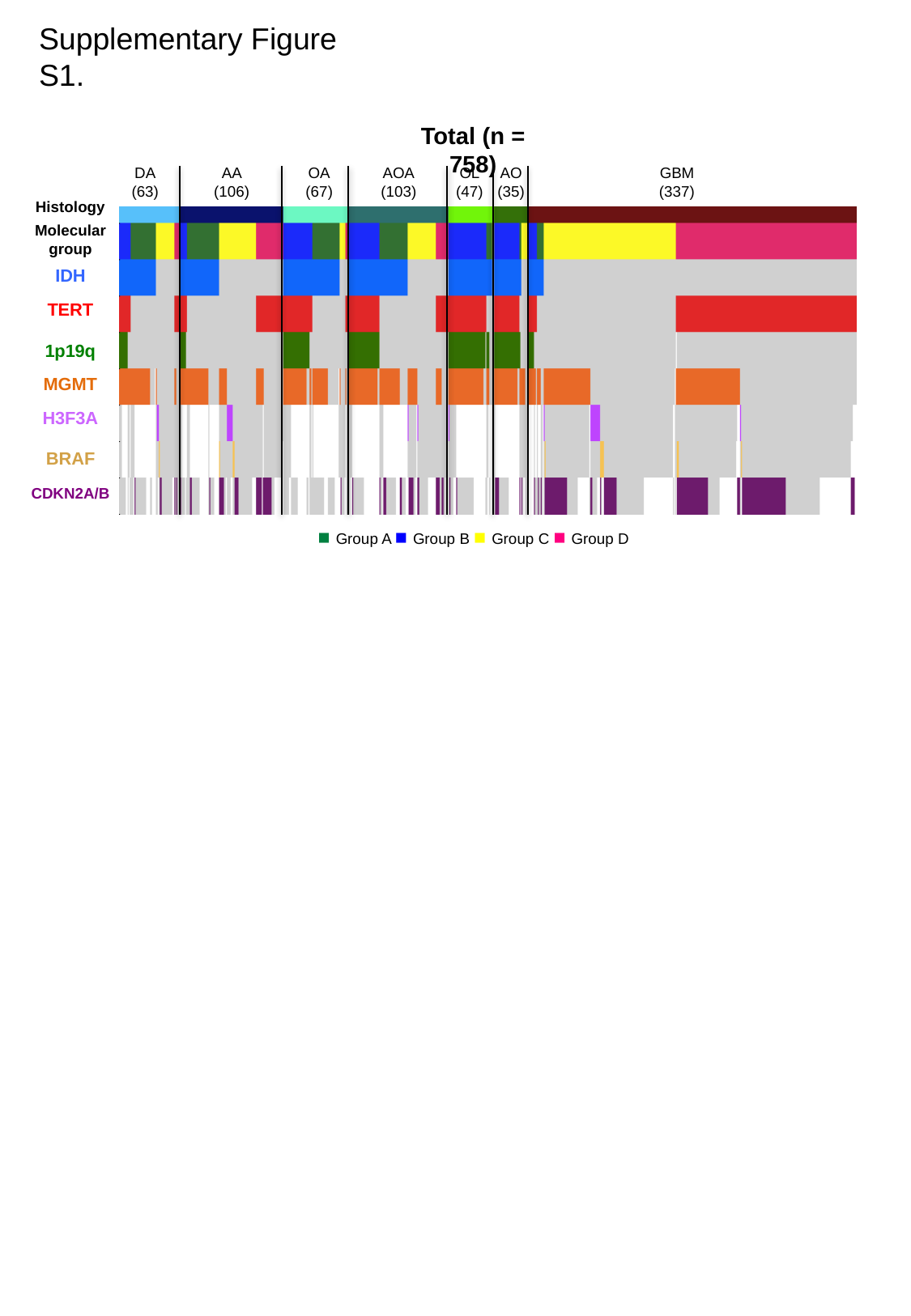

Supplementary Figure S1.
Total (n = 758)
DA
(63)
AA
(106)
OA
(67)
AOA
(103)
OL
(47)
AO
(35)
GBM
(337)
Histology
Molecular
group
IDH
TERT
1p19q
MGMT
H3F3A
BRAF
CDKN2A/B
■ Group A ■ Group B ■ Group C ■ Group D

## Slide 2
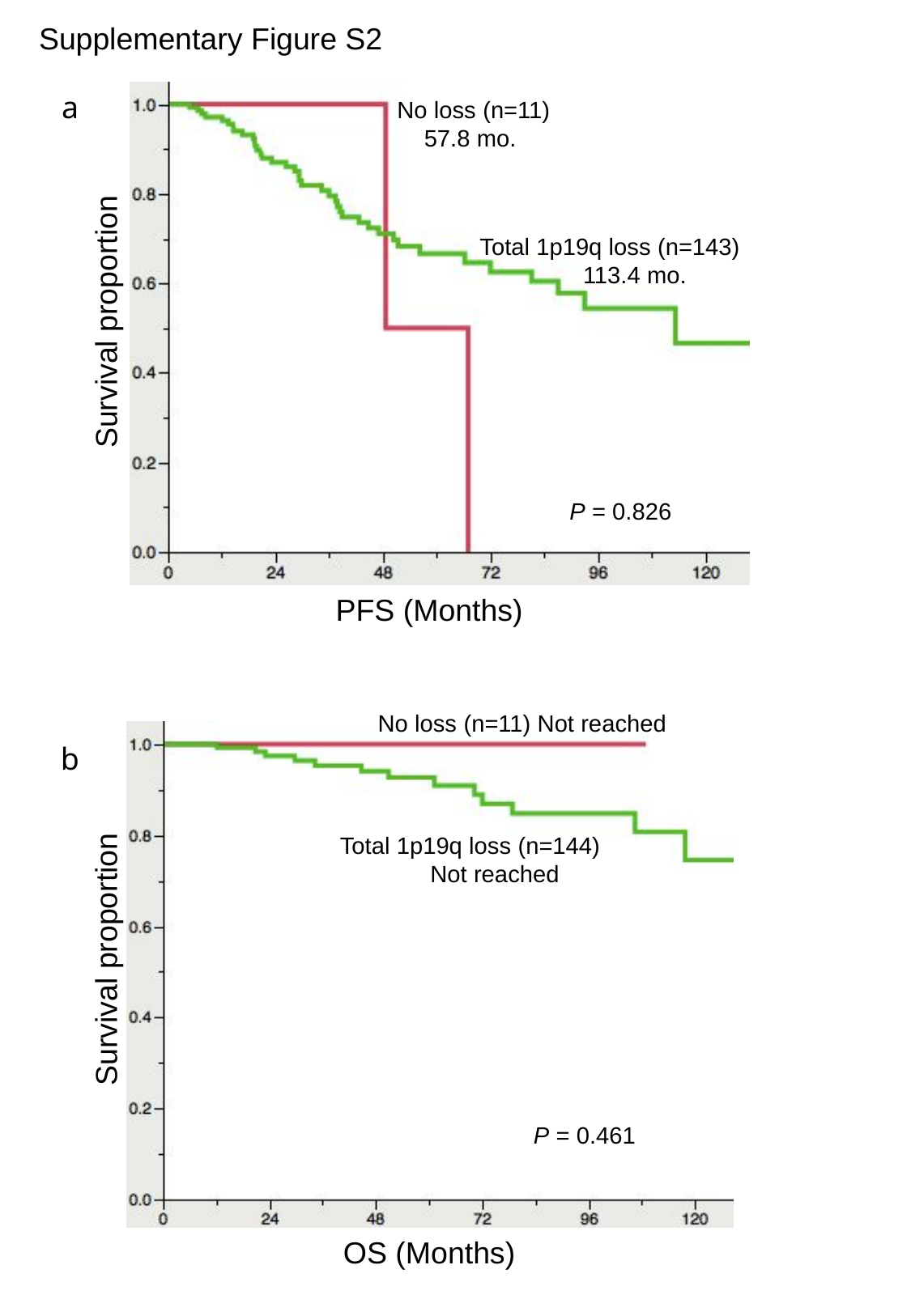

Supplementary Figure S2
a
No loss (n=11)
57.8 mo.
Total 1p19q loss (n=143)
113.4 mo.
Survival proportion
P = 0.826
PFS (Months)
No loss (n=11) Not reached
b
Total 1p19q loss (n=144)
Not reached
Survival proportion
P = 0.461
OS (Months)

## Slide 3
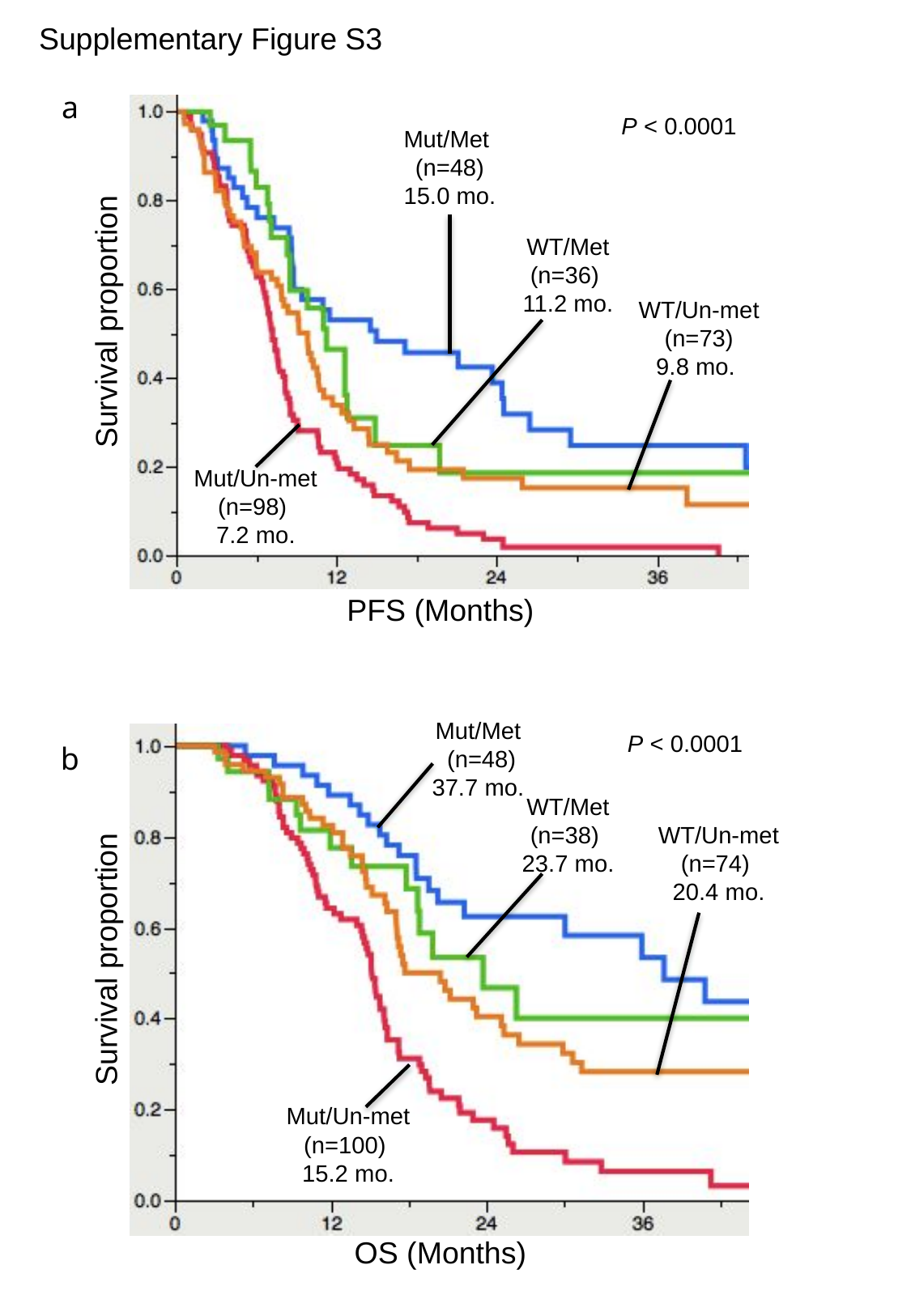

Supplementary Figure S3
a
P < 0.0001
Mut/Met
(n=48)
15.0 mo.
WT/Met
(n=36)
11.2 mo.
WT/Un-met
(n=73)
9.8 mo.
Survival proportion
Mut/Un-met
(n=98)
7.2 mo.
PFS (Months)
Mut/Met
(n=48)
37.7 mo.
P < 0.0001
b
WT/Met
(n=38)
23.7 mo.
WT/Un-met
(n=74)
20.4 mo.
Survival proportion
Mut/Un-met
(n=100)
15.2 mo.
OS (Months)

## Slide 4
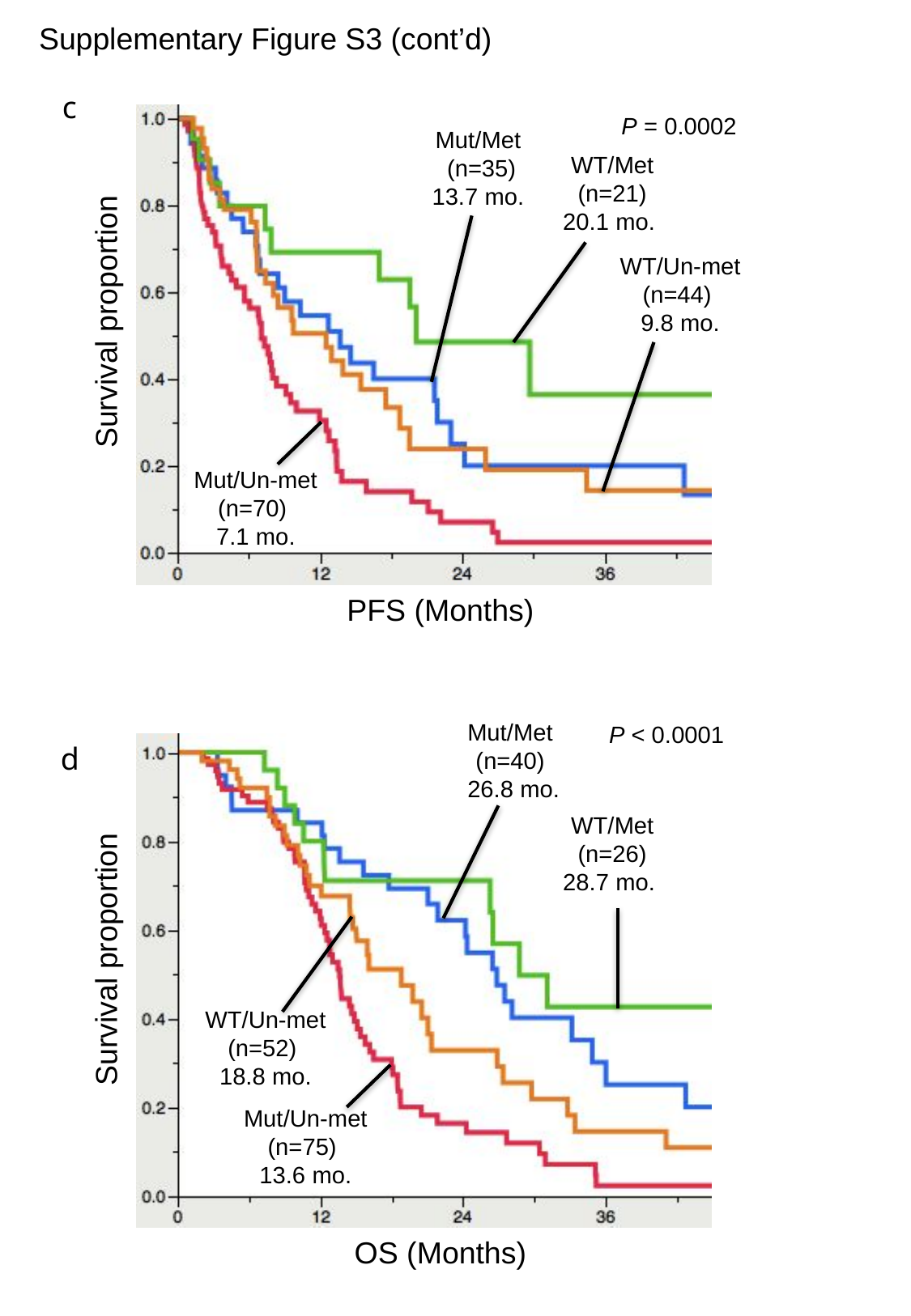

Supplementary Figure S3 (cont’d)
c
P = 0.0002
Mut/Met
(n=35)
13.7 mo.
WT/Met
(n=21)
20.1 mo.
WT/Un-met
(n=44)
9.8 mo.
Survival proportion
Mut/Un-met
(n=70)
7.1 mo.
PFS (Months)
Mut/Met
(n=40)
26.8 mo.
P < 0.0001
d
WT/Met
(n=26)
28.7 mo.
Survival proportion
WT/Un-met
(n=52)
18.8 mo.
Mut/Un-met
(n=75)
13.6 mo.
OS (Months)
